# Peptide-based immunotherapy of experimental autoimmune encephalomyelitis without anaphylaxis

**DOI:** 10.1002/eji.200737148

**Published:** 2007-12

**Authors:** Melanie D Leech, Chen-Yen Chung, Abigail Culshaw, Stephen M Anderton

**Affiliations:** University of Edinburgh, Institute of Immunology and Infection Research, School of Biological SciencesEdinburgh, UK

**Keywords:** Anaphylaxis, Autoimmunity, Multiple sclerosis, T cells, Tolerance

## Abstract

Administration of peptide antigens in tolerogenic form holds promise as a specific treatment for autoimmune and allergic disorders. However, experiments in rodent autoimmune models have highlighted the risk of anaphylaxis in response to systemic peptide application once the aberrant immune response is underway. Thus, mice with clinical signs of experimental autoimmune encephalomyelitis (EAE) or diabetes have been reported to suffer fatal anaphylaxis upon administration of native autoantigenic peptides. Clearly, this might represent a significant barrier to the use of synthetic peptides in the treatment of ongoing human autoimmune conditions. Here we describe the development of an altered peptide ligand (APL) engineered to prevent anaphylaxis (no antibody binding) whilst retaining the ability to silence pathogenic myelin-reactive T lymphocytes. Administration of the APL to mice with an ongoing anti-myelin immune response did not cause anaphylaxis, but led to complete protection from the subsequent induction of EAE and, when given during ongoing EAE, led to a rapid remission of clinical signs. The approach of removing antibody recognition whilst maintaining the desired functional effect (in this case T cell tolerance) may be of value in other situations in which there is a risk of triggering anaphylaxis with peptide-based drugs.

## Introduction

We and others have administered synthetic peptides containing T cell epitopes in soluble form to induce immune tolerance and prevent the development of various rodent models of autoimmune disease and several clinical trials are underway in humans [1–3]. Rodent studies have overwhelmingly focused on inducing tolerance in naive T cells, before exposure to the autoantigen in immunogenic form. In humans, the requirement is to switch off an autoaggressive response that is fully underway. Concern has been raised because the ongoing response can involve IgE antibodies capable of binding the synthetic peptides upon systemic administration, therefore leading to fatal anaphylactic responses in rodents [4–7]. This has particularly been shown in EAE, the T cell-driven mouse model of MS. Clearly, this is a potential complication that must be considered in the human setting. Indeed, a previous peptide-based trial in MS was halted because of evidence of developing hypersensitivity [8].

How can the risk of peptide-induced anaphylaxis be overcome? Based on studies using altered peptide ligand (APL), it is well established that T cell recognition of peptide epitopes is focused upon a few TCR contact residues [9]. We reasoned that anti-peptide antibody responses might similarly be focused on particular residues within the peptide. If the TCR contact and antibody contact residues were sufficiently diverse, we might be able to generate an APL that retained TCR binding, but not antibody binding, and therefore could induce tolerance but not anaphylaxis *in vivo* (TCR-binding is required to provide T cell tolerance). Here, we describe the use of such an APL to achieve profound tolerance in the face of an ongoing anti-myelin T cell response.

## Results and discussion

### Identification of antibody-binding residues with peptide 35–55 of MOG

For this study, we used EAE induced in C57BL/6 mice by immunization with the peptide 35–55 (p35–55) of myelin oligodendrocyte glycoprotein (MOG) [10]. Fatal anaphylaxis has been previously reported in this model when giving soluble p35–55 after EAE has developed [4], [6], and our early attempts to induce tolerance with the wild-type p35–55 were hampered by this effect (four of six mice requiring euthanasia).

**Figure 1:**
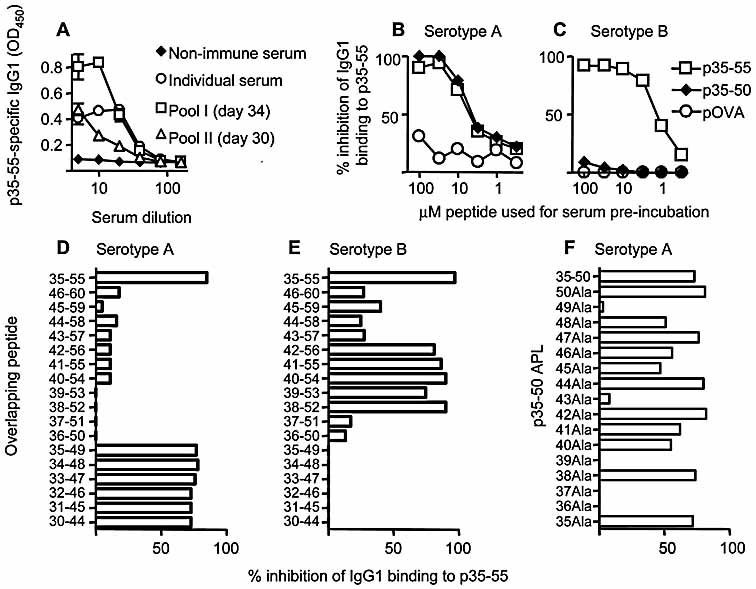
Identification of MOG35–50 (37Ala) as an APL that does not bind antibody. Binding of IgG1 to the p35–55 peptide was measured by ELISA (A–C). Pooled sera from mice that had undergone EAE (*i.e*. after immunization with p35–55 in CFA, together with administration of pertussis toxin, Pool I), or that had been immunized with p35–55 without EAE induction (*i.e*. with CFA, but without the use of pertussis toxin, Pool II), were titrated into a direct ELISA for p35–55 binding and the presence of IgG1 detected (A). Also shown is binding by an individual EAE serum sample and lack of binding by non-immune pooled syngeneic serum. Strongly binding individual sera (B, C) were pre-incubated with increasing doses of peptide (p35–55, p35–50, or ovalbumin 323–339 as a control) and inhibition of subsequent binding to p35–55 was calculated. Two serotypes were identified based on the ability of p35–50 to inhibit binding. Sera of type A or B were tested for blocking of p35–55-antibody binding by a single dose (30 µg/mL) of overlapping MOG peptides covering residues 30–60 (D, E). Sera of type A were preincubated with 30 µg/mL of p35–50, or APL thereof (F).

We therefore examined requirements for antibody-binding using sera from mice that had been immunized with p35–55. Surprisingly, we were unable to detect anti-p35–55 IgE in these sera, even after IgG-depletion (data not shown). However, significant titres of anti-p35–55 IgG1 were consistently observed (Fig. 1A). Using a competition ELISA, we were able to block binding of IgG1 to p35–55 by pre-incubation with the same peptide (Fig. 1B and C). We next utilized two panels of MOG peptides that we had generated for analysis of T cell activation in response to MOG. The first panel were overlapping 15mers covering the 35–55 sequence and shifting by one residue (*i.e*. 30–44, 31–45, etc, through to 46–60). The second panel were 16mer APL based on the 35–50 sequence with Ala substitutions at each individual residue (35Ala, 36Ala, etc). The 35–50 sequence was chosen because the core epitope for T cell recognition has been described as residues 40–48 [11]. These peptides were used in the competition ELISA to define the requirements for antibody binding (*i.e*. a positive signal in the ELISA, indicating that the peptide used for pre-incubation could not bind to the anti-p35–55 antibodies).

The first interesting observation using individual sera was that there were two serotypes; type A sera could bind to the wild-type p35–50 peptide (Fig. 1B), whereas type B sera could not (Fig. 1C). We next used the 15mer overlapping peptides to more precisely map the regions recognized by the type A and type B sera. Serotype A focused on residues 35–44 (Fig. 1D), whereas serotype B focused on residues 42–52 (Fig. 1E). Neither of these serotypes seemed to be dominant and as yet it is unclear what determines the serotype that an individual mouse will display.

We next tested the APL based on p35–50 for antibody binding. We could not use these APL to test for binding to type B sera, because these did not bind the native 35–50 peptide. Testing type A sera revealed five APL (36Ala, 37Ala, 39Ala, 43Ala and 49Ala) that failed to bind the sera (Fig. 1F).

### The 37Ala APL induces T cell tolerance and does not provoke anaphylaxis

**Figure 2:**
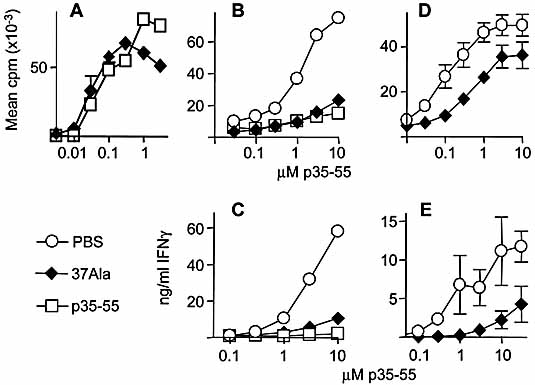
Tolerogenic properties of the MOG35–50 (37Ala) APL. Proliferation of a p35–55-reactive CD4^+^ T cell line was tested after culture with p35–55, or the p35–50(37Ala) APL (A). Mice were given a single 300-µg dose of soluble p35–55, or p35–50 (3Ala), or PBS alone intravenously, 7 days before immunization with p35–55 in CFA (CFA) (B, C). Ten days after immunization, draining inguinal and para-aortic lymph nodes were removed and tested for recall responses to p35–55 as measured by proliferation (B) or IFN-γ production (C). (D, E) Mice were immunized with p35–55 in CFA and 28 days later received soluble p35–50 (37Ala) or PBS as above. Seven days after peptide injection, spleens were removed and tested individually for recall proliferation (D) and IFN-γ production (E) to p35–55. Each tolerance experiment shown is one of two experiments giving consistent results; three mice per group were used.

To identify a peptide that did not cause anaphylaxis *in vivo* we chose to pursue the 37Ala APL because it involved alteration of a residue outwith the published core T cell epitope of 40–48 [11]. Furthermore, we found that this APL could stimulate p35–55-reactive T cells *in vitro*, with a dose response essentially identical to that of p35–55 (Fig. 2A). To test the ability of this peptide to induce T cell tolerance, we gave a single i.v. injection of the peptide in saline, either before or after immunization with p35–55 in CFA. The 37Ala APL proved as effective as the wild-type p35–55 peptide at inducing naive T cell tolerance *in vivo* when given in advance of p35–55 immunization (Fig. 2B and C).

We next gave an i.v. injection of the 37Ala APL, or a control APL, 38Ala that did bind anti-p35–55 antibodies (Fig. 1F), to mice that had been immunized 4 weeks previously with p35–55 in CFA and were sero-positive for anti-p35–55. Two of three mice that received the 38Ala APL showed anaphylaxis and were euthanised immediately. In contrast, none of the mice that received 37Ala showed signs of anaphylaxis. By sampling 7 days after this i.v. injection, we could therefore test whether these mice had become tolerant to p35–55. Compared with their PBS-treated counterparts, the mice that had received 37Ala, gave proliferative responses that were around 10-fold less sensitive to p35–55 (Fig. 2D) and IFN-γ responses that were approximately 100-fold less sensitive (Fig. 2E). These data suggested that a single dose of the 37Ala peptide had a marked tolerogenic effect on the ongoing anti-MOG T cell response but, crucially, did not cause anaphylaxis.

**Figure 3:**
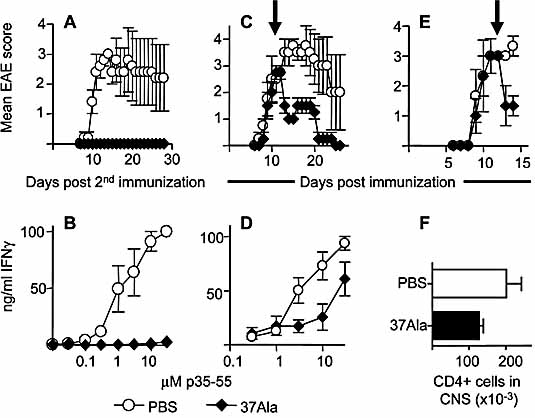
MOG35–50(37Ala) given after T cell priming protects mice from EAE. (A) Mice received a primary immunization with p35–55 in incomplete Freund's adjuvant supplemented with 60 µg CpG. Twenty-four days later mice received soluble p35–50(37Ala), or PBS, as above. Seven days after peptide injection, mice were given a secondary immunization with p35–55 in CFA to induce EAE. (B) Splenocytes were taken from EAE mice shown in (A) 28 days after the secondary immunization and tested individually for recall IFN-γ production in response to p35–55. (C–F) EAE was induced by a primary immunization with p35–55 in CFA. Twelve days later, mice received either PBS or p35–50(37Ala) i.v. (arrows) and effects on EAE were monitored (C, E). Individual splenocyte populations were tested for recall IFN-γ responses to p35–55 at day 26 (D). In the experiment shown in (E), mice were sacrificed on day 15 and numbers of infiltrating CD4^+^ cells in the CNS were assessed by FACS analysis. The experiment (four mice per group) shown in (A, B) is one of two that gave consistent results. Data in (C–F) are from two of four experiments (three to four mice per group) that gave consistent results. For (A) and (C), disease scores were significantly different between the PBS- and 37Ala-treated groups (*p* <0.001 in each case). Note: in these experiments, wild type p35–55 was not given in soluble form because our preliminary experiments showed that this peptide provoked anaphylaxis when injected after immunization.

Finally, we tested whether the 37Ala APL could influence the course of EAE. To test p35–55-immune mice before re-immunizing to induce EAE, we immunized with p35–55 in incomplete Freund's adjuvant supplemented with CpG oligonucleotide. This primary immunization protocol reliably induces strong anti-p35–55 T cell responses but not EAE, and allows EAE to develop subsequently in response to immunization with p35–55 in CFA with accelerated disease kinetics, indicating the presence of antigen-experienced cells generated by the primary immunization (Chung *et al.*, manuscript in preparation). We gave PBS, with or without the 37Ala APL, 24 days after the primary immunization. Seven days later, we immunized for a second time to induce EAE and found that the 37Ala-treated group were completely protected from disease (Fig. 3A). Upon *ex vivo* analysis, the mice that were protected by 37Ala treatment showed a complete absence of IFN-γ production (Fig. 3B) and IL-17 production (not shown) as well as markedly reduced proliferation (not shown) in recall responses to p35–55.

The above experiments showed that ongoing T cell response could be abrogated leading to protection from EAE upon subsequent secondary immunization. To test for an effect on the progression of active disease, we induced EAE with a primary immunization with p35–55 in CFA. We then gave a single i.v. dose of 300 µg 37Ala or PBS when clinical signs were in the ascendancy (day 12). Mice that received 37Ala showed a striking reduction in clinical signs 24 h later and this was maintained through the subsequent disease course (Fig. 3C). Analysis of splenocytes at day 26 revealed a reduced ability to produce IFN-γ in the 37Ala-treated group (Fig. 3D). The rapid effect that 37Ala had on EAE suggested that it may be acting on effector T cells within the target organ. To test this, we sampled CNS 3 days after giving 37Ala or PBS and found a reduced number of infiltrating CD4^+^ cells in the 37Ala-treated group (Fig. 3E and F). The protective effects of 37Ala on clinical EAE score were lost when a lower (200 µg) dose was administered, even though splenocytes from mice treated in this way showed reduced p35–55-induced recall responses (proliferation and IFN-γ production, data not shown). This suggests that for the peptide to work in active disease, a threshold level of peptide-MHC complexes needs to be achieved within the CNS.

Thus, in 37Ala we have identified a highly effective therapeutic APL that removes the risk of anaphylaxis. This allowed us to show that, the administration of peptide in soluble form could provoke profound tolerance to autoantigen either when given before a primary immunization, when given between primary and secondary immunizations and even when given at the height of active EAE. This latter observation is particularly pertinent, as we found no adverse clinical effects that could be attributed to excessive cytokine release from T cells in the CNS in response to the soluble peptide. The basic paradigm for peptide-induced tolerance in naive T cells is that antigen presentation by steady state DC leads to an abortive activation and T cell death due to insufficient survival signals [12]. It may well be that the tolerance induced here after immunization has a different basis, perhaps activation-induced cell death of differentiated effector T cells (a possibility supported by reduced CD4^+^ cell numbers in the CNS after peptide administration). Clarification of this will require further extensive analyses that are beyond the scope of this report.

In the clinical setting, repeated administration of peptides to MS patients has provoked anti-peptide IgE and IgG1 responses and hypersensitivity leading to the termination of that particular trial [8]. We could measure anti-p35–55 IgG1, but not IgE. It is unclear whether the anaphylaxis seen against p35–55 was the result of IgE at levels below detection, or because of the IgG1 that was evident. Although IgG1 is capable of binding to mast cells and could provide the anaphylactic trigger [13], a previous report using the same EAE model as we have used here has clearly implicated IgE and ruled-out IgG1 [6]. In potential human studies, where IgE might be more easily detected, this is likely to be the isotype that should be studied.

Many studies have used APL to alter T cell responses *in vitro* and *in vivo*, including the modulation of autoimmune models [14–16]. However, to our knowledge, this is the first study to show that we can use APL to define antibody-peptide binding, allowing peptide-based therapeutic immune tolerance in the absence of anaphylaxis.

## Concluding remarks

Here, we have addressed two key questions. First, can an ongoing autoimmune T cell response be silenced sufficiently to control disease (*i.e*. can peptides truly be used as treatments rather than prophylactically)? Second, can the previously identified risk of anaphylaxis be avoided by understanding the requirements for peptide interaction with antibody? The data presented here give a positive answer to both these questions. The use of peptides as small molecule drugs is a developing field [17] and anti-peptide anaphylaxis could be a real complication here. Therefore, variant peptides that can be shown to have the desired functional efficacy without binding to antibodies might have a more general relevance beyond immunotherapy.

## Materials and methods

### Mice, antigens, and immunizations

C57BL/6 mice were bred under specific pathogen-free conditions at the University of Edinburgh. The 6–8-week-old, sex-matched mice were used for all experiments. Peptides p35–55 and p35–50 of MOG and 323–339 of chicken ovalbumin were synthesized by the Advanced Biotechnology Centre, Imperial College (London, UK). Two panels of peptides were generated. The first panel were 15mers that shifted by one residue and covered MOG 30–44 to 46–60. The second panel were APL based on p35–50, with Ala substitutions at individual residues (35Ala, 36Ala, etc, through to 50Ala). These panels of peptides were synthesized by the laboratory of Professor D. Wraith, University of Bristol, UK. Unless otherwise stated, mice were immunized with 100 μg of p35–55 emulsified in complete Freund's adjuvant (CFA, Sigma, Poole, UK). A total of 100 µL of emulsion was injected s.c., 50 µL into each hind leg. Primed lymphoid populations were derived either from spleens, or from draining inguinal and para-aortic lymph nodes at the times indicated.

### pMOG-specific IgG1 ELISA

Microtitre plates were coated with 5 μg/mL p35–55 diluted in 0.05 M carbonate bicarbonate buffer (pH 9.6) prior to blocking nonspecific binding with 3% BSA in PBS. Sera were double diluted through eight consecutive dilutions from an initial 1 in 5 dilution in PBS-T. Bound antibody was detected using alkaline phosphatase labelled goat anti-mouse IgG1 (γ1 chain specific; Southern Biotech) and developed using pNNP substrate (Southern Biotech).

### Competition ELISA for inhibition of IgG1-p35–55 binding

Microtitre plates were coated with 5 μg/mL p35–55 and blocked as above. Sera were diluted to a final dilution of 1 in 40 in PBS-T (PBS with 0.1% Tween 20). Inhibition of antibody binding to p35–55 was tested by pre-absorption at 4°C for 30 min with graded doses of peptide, or with 30 μg/mL of either the p35–50 APL, or the overlapping peptides. Specificity of inhibition was determined by pre-incubation with peptide 323–339 of ovalbumin. Bound IgG1 was detected as above.

### Tolerance induction and assessment of lymphoid recall responses

Mice received 300 μg of peptide in 0.2 mL PBS (or PBS alone) i.v. at the indicated time before or after immunization with p35–55. Lymphoid-cell suspensions were cultured in 96-well flat-bottom microtitre plates (Becton Dickinson, Oxford, UK) at 6 × 10^5^ lymph node cells/well, or 8 × 10^5^ splenocytes/well, using X-vivo 15™ serum-free medium (BioWhittaker, Maidenhead, UK) supplemented with 2 mM L-glutamine and 5 × 10^–5^ M 2-ME (all from Invitrogen Life Technologies, Paisley, UK). Cultures were stimulated with a dose range of p35–55 for 48 h prior to addition of [^3^H]thymidine (0.5 μCi/well) (Amersham, Amersham, UK). After further 18 h, cultures were harvested and thymidine incorporation was measured using a liquid scintillation β-counter (LKB Wallac, Turku, Finland). Results are expressed as mean cpm of triplicate cultures. Supernatants from similar 72-h cultures were tested for p35–55-induced production of IFN-γ and IL-17 by ELISA.

### p35–55-reactive T cell line

The PP.TCL CD4^+^ T cell line was generated using repeated restimulation and expansion cycles as described previously [18]. Proliferation assays were performed using flat-bottom 200 μL microtitre wells (Becton Dickinson). T cells (2 × 10^4^/well) were cultured with irradiated (30 Gy) syngeneic splenocytes (3 × 10^5^/well) in the presence or absence of peptide antigen for a total of 72 h. Cultures were pulsed for the final 16 h with [^3^H]thymidine and incorporation measured as above.

### Induction and assessment of EAE

EAE was induced using a previously described protocol [19]. In some experiments this was modified as follows. Mice were first immunized in one hind leg with 50 µg of p35–55 in 50 µL incomplete Freund's adjuvant supplemented with 60 µg of CpG oligonucleotide (MWG Biotech, London, UK). At the indicated times, mice then received either 300 µg of the p35–50(37Ala) in PBS or PBS alone intravenously. EAE was then induced by a second immunization with 100 µg p35–55 in 50 µL CFA into the other hind leg. Mice also received 200 ng pertussis toxin (Health Protection Agency, Dorset, UK) i.p. in 0.5 mL PBS on the same day and 2 days later. Group sizes were 4–6 per treatment group.

Clinical signs of EAE were assessed using the following scoring index: 0, no signs; 1, flaccid tail: 2, impaired righting reflex and/or impaired gate; 3, partial hind leg paralysis; 4, total hind leg paralysis; 5, hind and fore leg paralysis; 6, moribund or dead. Differences in total disease burden between groups were determined using the Mann-Whitney U-test. CNS mononuclear cell samples (from brain and spinal cord) were prepared and stained for CD4-expression as described previously [19].

## Abbreviations:

APL: altered peptide ligand
MOG: myelin oligodendrocyte glycoprotein

## References

[1] Anderton SM (2001). Peptide-based immunotherapy of autoimmunity: a path of puzzles, paradoxes and possibilities. Immunology.

[2] Larche M, Wraith DC (2005). Peptide-based therapeutic vaccines for allergic and autoimmune diseases. Nat. Med..

[3] Prakken BJ, Samodal R, Le TD, Giannoni F, Yung GP, Scavulli J, Amox D (2004). Epitope-specific immunotherapy induces immune deviation of proinflammatory T cells in rheumatoid arthritis. Proc. Natl. Acad. Sci. USA.

[4] Pedotti R, Mitchell D, Wedemeyer J, Karpuj M, Chabas D, Hattab EM, Tsai M (2001). An unexpected version of horror autotoxicus: anaphylactic shock to a self-peptide. Nat. Immunol..

[5] Pedotti R, Sanna M, Tsai M, DeVoss J, Steinman L, McDevitt H, Galli SJ (2003). Severe anaphylactic reactions to glutamic acid decarboxylase (GAD) self peptides in NOD mice that spontaneously develop autoimmune type 1 diabetes mellitus. BMC immunology.

[6] Smith CE, Eagar TN, Strominger JL, Miller SD (2005). Differential induction of IgE-mediated anaphylaxis after soluble vs. cell-bound tolerogenic peptide therapy of autoimmune encephalomyelitis. Proc. Natl. Acad. Sci. USA.

[7] Liu E, Moriyama H, Abiru N, Miao D, Yu L, Taylor RM, Finkelman FD, Eisenbarth GS (2002). Anti-peptide autoantibodies and fatal anaphylaxis in NOD mice in response to insulin self-peptides B:9–23 and B:13–23. J. Clin. Invest..

[8] Kappos L, Comi G, Panitch H, Oger J, Antel J, Conlon P, Steinman L (2000). Induction of a non-encephalitogenic type 2 T helper-cell autoimmune response in multiple sclerosis after administration of an altered peptide ligand in a placebo-controlled, randomized phase II trial. The altered peptide ligand in relapsing MS study group. Nat. Med..

[9] Sloan-Lancaster J, Allen PM (1996). Altered peptide ligand-induced partial T cell activation: molecular mechanisms and role in T cell biology. Annu. Rev. Immunol..

[10] Mendel I, Kelero de Rosbo N, Ben-Nun A (1995). A myelin oligodendrocyte glycoprotein peptide induces typical chronic experimental autoimmune encephalomyelitis in H-2(B) mice - fine specificity and T-cell receptor V-beta expression of encephalitogenic T-cells. Eur. J. Immunol..

[11] Mendel I, Kelero de Rosbo NK, Ben-Nun A (1996). Delineation of the minimal encephalitogenic epitope within the immunodominant region of myelin oligodendrocyte glycoproteDiverse VP gene usage by T cells recognizing the core epitope encephalitogenic for T cell receptor V beta(b) and T cell receptor V beta(a) H-2(b) mice. Eur. J. Immunol..

[12] Hochweller K, Sweenie CH, Anderton SM (2006). Immunological tolerance using synthetic peptides - Basic mechanisms and clinical application. Curr. Molec. Med..

[13] Strait RT, Morris SC, Yang M, Qu X-W, Finkelman FD (2002). Pathways of anaphylaxis in the mouse. J. Allerg. Clin. Immunol..

[14] Anderton SM, Kissler S, Lamont AG, Wraith DC (1999). Therapeutic potential of TCR antagonists is determined by their ability to modulate a diverse repertoire of autoreactive T cells. Eur. J. Immunol..

[15] Nicholson LB, Greer JM, Sobel RA, Lees MB, Kuchroo VK (1995). An altered peptide ligand mediates immune deviation and prevents autoimmune encephalomyelitis. Immunity.

[16] Margot CD, Ford ML, Evavold BD (2005). Amelioration of established experimental autoimmune encephalomyelitis by an MHC anchor-substituted variant of proteolipid protein 139–151. J. Immunol..

[17] Gentilucci L, Tolomelli A, Squassabia F (2006). Peptides and peptidomimetics in medicine, surgery and biotechnology. Curr. Medicin. Chem..

[18] Sweenie CH, Mackenzie KJ, Rone-Orugboh A, Liu M, Anderton SM (2007). Distinct T cell recognition of naturally processed and cryptic epitopes within the immunodominant 35–55 region of myelin oligodendrocyte glycoprotein. J. Neuroimmunol..

[19] McGeachy MJ, Stephens LA, Anderton SM (2005). Natural recovery and protection from autoimmune encephalomyelitis: Contribution of CD4^+^CD25^+^ regulatory cells within the central nervous system. J. Immunol..

